# Integrase inhibitors effective against human T-cell leukemia virus type 1

**DOI:** 10.1186/1742-4690-8-S1-A34

**Published:** 2011-06-06

**Authors:** Muhammad E Seegulam, Lee Ratner

**Affiliations:** 1 Division of Molecular Oncology, Washington University, St Louis, MO, 63110, USA

## 

Drugs targeting the viral enzyme integrase have been in use for several years as part of the treatment regimen for patients with Human Immunodeficiency Virus Type 1 (HIV-1) and similar classes of compounds have been shown to inhibit Human T-Cell Leukemia Virus Type 1 (HTLV-1) integration in vitro. The current study shows that the clinically approved HIV-1 integrase inhibitor, Raltegravir, as well as the more recent diketo acid derivative, MK-2048, are active inhibitors of HTLV-1 infection in vitro. These agents were effective in inhibiting cell-free and cell-to-cell transmission of HTLV-1 in lymphoid and non-lymphoid cells. The drugs also inhibited HTLV-1 immortalization of human peripheral blood mononuclear cells. A novel adaptation of the Alu assay for viral integration was used to show that the drugs inhibit viral integration without affecting reverse transcription. These data support the administration of Raltegravir and other integrase inhibitors as treatments for patients with HTLV-1 associated diseases.

